# Taxonomic differences of gut microbiomes drive cellulolytic enzymatic potential within hind-gut fermenting mammals

**DOI:** 10.1371/journal.pone.0189404

**Published:** 2017-12-27

**Authors:** Emma C. L. Finlayson-Trick, Landon J. Getz, Patrick D. Slaine, Mackenzie Thornbury, Emily Lamoureux, Jamie Cook, Morgan G. I. Langille, Lois E. Murray, Craig McCormick, John R. Rohde, Zhenyu Cheng

**Affiliations:** 1 Department of Microbiology and Immunology, Dalhousie University, Halifax, Nova Scotia, Canada; 2 Department of Pharmacology, Dalhousie University, Halifax, Nova Scotia, Canada; USDA Forest Service, UNITED STATES

## Abstract

Host diet influences the diversity and metabolic activities of the gut microbiome. Previous studies have shown that the gut microbiome provides a wide array of enzymes that enable processing of diverse dietary components. Because the primary diet of the porcupine, *Erethizon dorsatum*, is lignified plant material, we reasoned that the porcupine microbiome would be replete with enzymes required to degrade lignocellulose. Here, we report on the bacterial composition in the porcupine microbiome using 16S rRNA sequencing and bioinformatics analysis. We extended this analysis to the microbiomes of 20 additional mammals located in Shubenacadie Wildlife Park (Nova Scotia, Canada), enabling the comparison of bacterial diversity amongst three mammalian taxonomic orders (Rodentia, Carnivora, and Artiodactyla). 16S rRNA sequencing was validated using metagenomic shotgun sequencing on selected herbivores (porcupine, beaver) and carnivores (coyote, Arctic wolf). In the microbiome, functionality is more conserved than bacterial composition, thus we mined microbiome data sets to identify conserved microbial functions across species in each order. We measured the relative gene abundances for cellobiose phosphorylase, endoglucanase, and beta-glucosidase to evaluate the cellulose-degrading potential of select mammals. The porcupine and beaver had higher proportions of genes encoding cellulose-degrading enzymes than the Artic wolf and coyote. These findings provide further evidence that gut microbiome diversity and metabolic capacity are influenced by host diet.

## Introduction

The microbiome supports animal digestion by detoxifying and breaking down indigestible compounds [1]. There is accumulating evidence that microbial enzymes responsible for these processes are highly conserved across species, and strongly influence overall composition of the microbiome [2]. Intensive study of the human gut microbiome provides a framework for investigating the phylogenetic diversity of wildlife gut microbiomes [2–14]. Similar to what has previously been observed in human microbiomes [15], the gut microbial diversity of beavers [10], ruminant mammals [14], and giant pandas [16], is strongly influenced by diet. This provides ample rationale for exploring the gut microbiome as a genetic repository of novel enzymes involved in processing diverse dietary components. Animal gut microbiomes provide a potentially rich source of novel microbial genes that can be leveraged for bioengineering applications, including breakdown of complex plant-derived materials [17].

Lignocellulose, a component of the plant cell wall, is an attractive low-cost substrate for biofuel production via microbial fermentation [10]. Before lignocellulose can be fermented, it must be broken down into component sugars by cellulases, hemicellulases, and various debranching enzymes [18]. The porcupine, *Erethizon dorsatum*, is an herbivore that feeds on lignified plants, coniferous (preferred) and deciduous cambium (inner bark), and flowers [19]. As a hindgut fermenter, the porcupine has an enlarged cecum where microbial digestion is largely confined [20]. Because the primary diet of porcupines is lignified plant material, we hypothesized that the porcupine microbiome would be replete with enzymes required to degrade lignocellulose.

The primary objective of this study, completed by undergraduate students of the Dalhousie iGEM team, was to characterize the gut microbiome of the porcupine along with 20 other species (within the Rodentia, Carnivora, and Artiodactyla orders) from the Shubenacadie Wildlife Park in Nova Scotia, Canada. All species were profiled using 16S rRNA gene sequencing. Mammals in the same order had more similar microbiome profiles compared to those with differing taxonomic classification. Metagenomic shotgun sequencing was conducted on select representative herbivores (porcupine, *Castor canadensis* (beaver)) and carnivores (*Canis latrans* (coyote), *Canis lupus arctos* (Arctic wolf)). In these four selected mammals, dramatic bacterial taxonomic differences were detected using both 16S and metagenomic sequencing methods. Importantly, the genes encoding cellulolytic enzymes were significantly enriched in porcupine and beaver microbiomes. Taken together, these findings confirm that the microbiomes of hind-gut fermenters are a rich source of cellulose-degrading enzymes.

## Methods

### Data availability

Quality controlled and processed metagenomic and 16S rDNA sequence data are available through the Sequence Read Archive (NCBI) at accession number SRP115632 and SRP115643 respectively.

### Animal containment and diet

All 21 animals were sampled from Shubenacadie Wildlife Park. Animals lived in outdoor pens modeled after their natural habitats. For instance, roaming animals, such as deer and elk, were held in large pastures with unlimited access to grass. Animals were co-housed regardless of whether they were born in captivity or rescued from the wild. All animals received diets that met their species-specific requirements (Table 1).

**Table 1:** Diets of the 21 mammals sampled from Shubenacadie Wildlife Park. Presented is the animal common name, the daily diet, and the speciality food stuffs required to meet dietary requirements.

Animal	Daily Diet	Speciality Foods (Not provided daily)
Arctic fox	100 g Pacifica dry dog food, 100 g wild prairie dog food,	250 g whole rabbit, hard-boiled egg, 25 g berries and fruit, 200 g ground chicken, 200 g lean horse
Arctic wolf	1.8 kg canine diet, 2 cups of large breed dog food	2.2 kg ground chicken, hard-boiled egg, 2 kg whole rabbit, and 1.25 kg of beef knuckles
Beaver	200 g alfalfa, 250 g rabbit chow, 35 g chopped carrots, 35 g chopped apple, 35 g chopped cabbage, 35 g chopped yam, 35 g chopped broccoli	unlimited branches (alder, maple, birch, and poplar)
Black bear	2 kg canine plus large breed adult, hard-boiled egg, 75 g berries and fruit, 100 g of each; carrots, yams, and apples	2 kg canine diet, fish oil, 2 kg whole herring, 75 g of berries and fruit
Elk	Browser pellets, Purina deer pellets, Timothy hay, salt block	pasture grazing
Fisher	300 g small carnivore diet, fish oil	300 g ground chicken, 25 g berries or fruit, 300 g lean horse meat, 300 g whole rabbit
Mink	100 g otter mixture	80 g whole turkey, 20 g berries or fruit
Moose	Mazuri moose maintenance-free choice @ 100%, 1 kg cut-up browse (Birch, Willow, Alder, Maple, Aspen and Balsam Fir) leaves and small branches—1/4" or less in diameter), 20 g equine plus supplement, salt block	pasture grazing
Pine martin	150 g small carnivore diet	150 g ground chicken, 25 g berries or fruit, 150 g turkey
Porcupine	250 g rabbit chow, 100 g alfalfa, 15 g of each; chopped carrot, chopped apple, potato, fruit and berries, salt block	free access to softwood trees, antler, free access to sod
Raccoon	150 g Pacifica dry food, 150 g prairie dry dog food, 40 g berries and fruit, 40 g chopped apple	hard-boiled egg, 250 g small carnivore diet
Red deer	Free choice; Browser pellets, deer pellets, Timothy hay, salt block	free range
Striped skunk	35 g dry dog food, 35 g prairie dog food, 20 g apple, 20 g fruit or berries	hard-boiled egg
Swift fox	75 g dry dog food, 75 g prairie dog food, 25 g berries or fruit	150 g whole rabbit, 150 g small carnivore diet, hard-boiled egg 150 g ground chicken, 150 g lean horse
Coyote	800 g canine diet, 1 cup large breed dry dog food, fish oil	900 g chicken necks, hard-boiled egg, 1 kg whole rabbit, 750 g beef knuckles, vitamin supplement

### DNA extraction and sequencing

Fecal samples from 21 different mammals at Shubenacadie Wildlife Park were collected by park staff and stored at -80˚C [21]. DNA was extracted using a Mobio PowerFecal® extraction kit (QIAGEN, Germany) according to the manufacturer's protocol. DNA purity was assessed using a Nanodrop spectrophotometer (NanoDrop Technologies Inc., USA). DNA samples were submitted for 16S rRNA gene sequencing at the Integrated Microbiome Resource (IMR) at Dalhousie University. 16S rRNA sequencing primers (Table 2), provided by the IMR, were modified from the primers used by Comeau *et*. *al*. to improve compatibility with the Illumina platform [22]. Variable regions V6-V8 of the bacterial 16S rRNA gene were amplified from all purified DNA samples and sequenced on an Illumina MiSeq using paired-end 300 bp sequencing [23]. For metagenomic shotgun sequencing, extracted DNA from coyote, porcupine, Arctic wolf and beaver samples was PCR-amplified using primers from Nextera following the Nextera XT (Illumina) protocol. Then, sequencing libraries were prepared from purified PCR products (1 ng) using the Nextera XT Library Preparation Kit (Illumina). Libraries were cleaned-up and normalized using the Just-a-Plate 96 PCR Purification and Normalization Kit (Charm Biotech). Complete libraries were then pooled and sequenced in a portion of a 300+300 bp Pair-End MiSeq run (Illumina 600-cycle v3 kit).

**Table 2:** List of oligonucleotides used for 16S rDNA amplification.

Name of Primer	Sequence	Description
Forward (V6)	ACGCGHNRAACCTTACC	Forward Primer for 16S rDNA gene
Reverse (V8)	ACGGGCRGTGWGTRCAA	Reverse Primer for 16S rDNA gene

### 16S sequencing data analysis

Analysis of 16S rRNA sequence data was performed as previously described using Microbiome Helper [23]. Samples from each of the 21 mammals were sequenced only once, with exceptions in the beaver, porcupine, Arctic wolf and coyote which were sequenced in biological triplicate. Raw data in FASTQ format from Illumina sequencing was analyzed for quality via FASTQC. Paired-end reads were stitched together using PEAR [24]. Stitched reads were filtered for quality (q-score >30 over at >90% of the read) and to ensure forward and reverse primers matched using FASTX-toolkit and BBMAP. FASTQ files were converted to FASTA format and all sequences containing a variable nucleotide ‘N’ were removed. Chimeric reads were removed using VSEARCH [25]. Taxonomic assignments were made by matching sequences into Operational Taxonomic Units (OTUs) with 97% sequence identity and comparing to the GreenGenes 16S rRNA database using QIIME [26–27]. Samples were normalized for differences in sequencing depth using DeSeq2’s negative binomial distribution [28] and principle coordinate analysis was performed using UniFrac beta-diversity through QIIME [29].

KEGG Orthologs (KO) [30–32] were inferred using PICRUSt to estimate functional-gene profiles from the triplicate porcupine, beaver, coyote, and Arctic wolf triplicate 16S sequencing data [33]. Differences in KO relative abundances across species were analyzed using STAMP, and subjected to an ANOVA analysis with Benjamini-Hochberg FDR correction to generate the predicted mean proportion of sequences and the box-plots [34].

### Metagenomic sequencing data analysis

Raw sequences were inspected for overall quality using FASTQC and then stitched using the program PEAR [24], followed by screening for potential contaminant sequences from human and spiked-in PhiX using BowTie2 [35]. We opted to only use stitched reads to avoid artificial annotation due to short read fragments, and to ensure higher quality annotation. After removal of contaminant data, the software program Trimmomatic was used with default quality cut offs based on FASTQC inspection [36]. MetaPhlAn2 was used to screen the data for taxonomy, and assign reads to bacterial species [37]. Following taxonomic assignment, DIAMOND and HUMAnN1 were used to assign functionality and metabolic profiles to the sequenced metagenomes. DIAMOND was used to perform the initial Pre-HUMAnN1 search against the KEGG database with a default e-value cutoff of 0.001 (stringent selection for protein matches) [38]. The HUMAnN1 software tool was used to get a functional profile of the metagenomic sequences which was then converted to STAMP format [39]. STAMP was used to perform an ANOVA analysis with Benjamini-Hochberg FDR correction and to generate box plots for comparison to the PICRUSt analysis from the 16S rRNA sequencing above. The data output from MetaPhlAn2 was visualized using GraPhlAn, after conversion of the MetaPhlAn2 output data with the Export2GraPhlAn scripts. Summary heat maps of the HUMAnN1 output were generated using Morpheus hierarchal clustering with a one minus Pearson correlate matrix and an average linkage method [40]. All four animals were sequenced in triplicate.

HUMAnN2 was run on the metagenomic dataset to confirm the HUMAnN1 results and to identify taxa likely contributing to the gene families of interest in each sample [38]. HUMAnN2 was run in uniref-50 mode using the same KEGG database as used for HUMAnN1 analysis. HUMAnN2 gene family abundance data was analyzed using a two-way ANOVA with the Holm-Sidak multiple test correction.

## Results

### Captive mammals display overlapping microbiome composition

Illumina sequencing of the 16S rRNA gene (V6-8 region) for the 21 Shubenacadie Wildlife Park mammals produced a total of 1,002,901 paired-end overlapping reads (Table 3). After merging paired-end reads and filtering for sequences longer than 400 base pairs (90% read-quality of >30), samples were left with at least 84% of their initial number of sequences. The number of OTUs identified per sample at 97% sequence identity when clustering ranged from 786 (Skunk) to 5,310 (Red Deer). Alpha rarefaction curves confirmed OTU saturation for most of the sequences, although the porcupine, moose, and red deer samples appeared to have less saturated sequencing than the others (S1 Fig).

**Table 3:** Summary of profiled species and 16S rRNA sequencing details. Presented is the common animal name, the scientific animal name, the total number of sequences, the number of OTUs per sample, number of unique OTU’s per sample, and the number of remaining sequences after stitching and quality filtering.

Host Animal	*Scientific Name*	OTU’s identified per Sample	Unique OTU’s	Total Sequences	Sequences after Pairing/Quality Filtering
Red Fox	*Vulpes vulpes*	970	283	50,870	43,120
Domestic Rabbit	*Oryctolagus cuniculus*	1,353	995	39,559	33,598
Otter	*Lontra canadensis*	881	425	61,242	52,578
Coyote	*Canis latrans*	1,275	625	50,496	42,765
Mink	*Neovision vision*	1,082	660	60,591	52,126
Skunk	*Mephitis mephitis*	786	328	55,787	47,833
Raccoon	*Procyon lotor*	850	400	44,213	37,857
Black Bear	*Ursus americanus*	1,152	622	49,409	42,604
Porcupine	*Erethizon dorsatum*	3,350	2,894	20,933	17,599
Elk	*Cervus canadensis*	2,144	1,104	19,599	16,546
Fisher	*Pekania pennanti*	793	308	64,867	56,003
Arctic Fox	*Vulpes lagopus*	2,296	1,178	55,170	46,954
Snowshoe Hare	*Lepus americanus*	1,560	1,157	64,785	54,949
Marten	*Martes americana*	814	316	82,374	70,689
Beaver	*Castor canadensis*	2,155	1,920	54,564	46,209
Swift Fox	*Vulpes velox*	923	172	47,605	40,081
Arctic Wolf	*Canis lupus subs*. *arctos*	1,354	657	34,253	28,954
Red Deer	*Cervus elaphus*	5,310	3,553	24,662	21,805
Moose	*Alces alces*	3,997	2,771	27,640	23,336
Timber Wolf	*Canis lupus*	917	314	45,391	38,354
Ground Hog	*Marmota monax*	2,301	1,640	47,891	41,039

**Fig 1:**
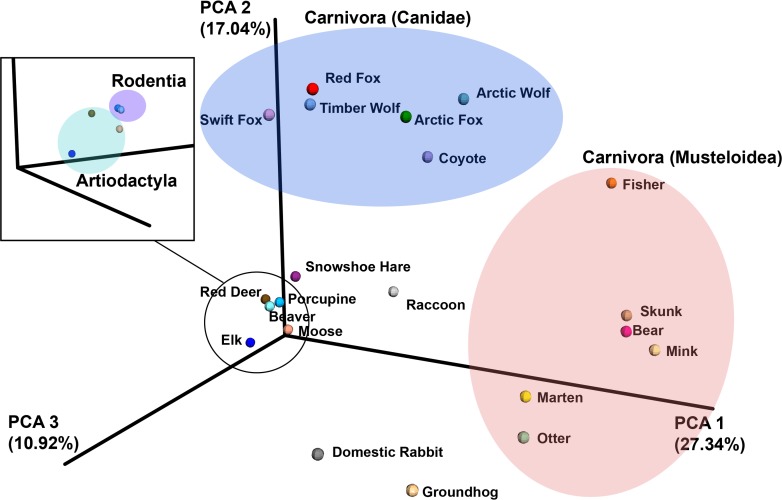
A 3-dimensional principal component analysis plot of the weighted UniFrac beta-diversity for 21 profiled species. The mammals belonging to a taxonomic order, indicated with **bold** font, are encircled to show the order’s space on the plot. The zoomed-in black box depicts a section of the graph rotated in 3D space to show the slight, but present separation between the two taxonomic orders Rodentia and Artiodactyla.

The 21 mammalian microbiomes were then compared using weighted unifrac beta-diversity analysis of the 16S rRNA sequencing data (Fig 1). The principal component analysis showed that mammals in the same order (Carnivora, Rodentia, Artiodactyla, etc.) had more similar microbiomes compared to those from different orders. The order Carnivora was observed twice in the beta-diversity plot representing two different clusters of Carnivora: the *Canidae* family (including foxes, wolves, and coyotes), and the *Musteloidae* superfamily (including the fisher, skunk, otter, mink, marten, and bear) [41–42]. The two distinct clusters indicated that the microbiomes of the Carnivora (super)families were divergent, and warranted separate locations on the beta-diversity plot. Next, the specific composition of the microbiome was analyzed through the 16S data of the sampled animals.

**Fig 2:**
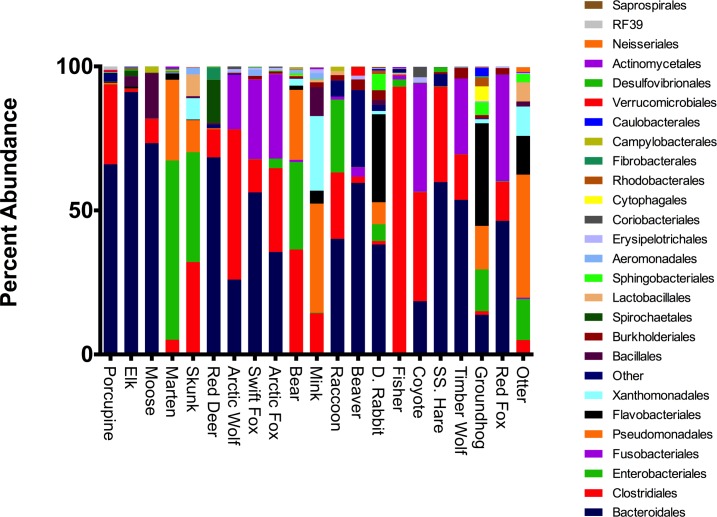
Microbiome analysis of all sequenced mammals shows great bacterial diversity between samples with some overlap in taxonomic orders. Taxonomic relative abundances of 16S rRNA data collapsed to sequences of indicated bacterial orders were identified from fecal samples of 21 mammals. All sequences under 0.01% abundance were clustered into the Other category.

Bacteroidales was the most prominent order isolated from both the porcupine and the beaver were (66.0%, 59.6% respectively; Fig 2). The next most abundant order in the porcupine was Clostridiales (27.7%), whereas the next most abundant order in the beaver was Verrucomicrobials (3.1%) (Fig 2). A significant number of bacteria were unassigned in both the porcupine and beaver datasets (2.8%, 26.8%), suggesting either the presence of novel species within these hosts and/or poor quality of reads (Fig 2). Like the porcupine and beaver, the microbiomes from the Arctic wolf, swift fox, Arctic fox, coyote, timber wolf, and red fox contained a large percentage of Bacteroidales (26.0%, 56.3%, 35.6%, 18.5%, 53.7% and 46.4%) and Clostridiales (52.2%, 11.5%, 29.1%, 37.5%, 26.3% and 37.0%), while containing two other abundant orders: Fusobacteriales (19.0%, 27.6%, 29.3%, 37.5%, 26.3%, and 36.9%) and Burkholderiales (0.4%, 1.2%, 0.7%, 0.2%, 3.7% and 2.0%; Fig 2). The most abundant orders found in the Artiodactyla order (Elk, Moose, and Red Deer) were: Bacteroidales (58.1%, 36.5% and 26.3% respectively), Bacillales (3.6%, 15.6%, and 0.5%) and Spirochaetales (2.0%, 0.1%, and 15.1%; Fig 2). In the Artiodactyla order (Elk, Moose, and Red Deer), Clostridiales was less abundant (0.5%, 2.9% and 5.1%; Fig 2). For the mammals in the Musteloidae order (Marten, Skunk, Mink, Fisher, and Otter), the most abundant bacterial order was Clostridiales (5.1%, 32.1%, 14.2%, 92.8%, and 5.0%) followed by Pseudomonadales (28.0%, 11.1%, 37.8%, 0.5%, and 42.8%) and Enterobacteriales (62.3%, 38.1%, 0.2%, 2.6%, and 14.2%; Fig 2). Finally, there were five mammals—the raccoon, domestic rabbit, snowshoe hare, bear, and groundhog—that did not group into the Carnivora, Rodentia, or Artiodactyla orders. Bacteroidales was the most abundant bacterial order in the raccoon, domestic rabbit, and snowshoe hare (40.1%, 38.1%, and 59.8% respectively), whereas Clostridiales was most abundant in the bear (36.3%), and Flavobacteriales was most abundant in the groundhog (36.6%; Fig 2). Clostridiales was also abundant in the raccoon and snowshoe hare microbiomes (23.0%, and 33.1%, respectively), but scarce in the domestic rabbit and groundhog (1.2%, and 1.3%, respectively; Fig 2). The complete taxonomic assignments for 16S rRNA sequencing datasets can be found in S1 Table.

Further taxonomic characterization was conducted by 16S sequencing, in biological triplicate, on fecal samples from a subset of four mammals: the beaver and the porcupine (cellulose-consuming mammals), and the coyote and the Arctic wolf (carnivorous mammals). Alpha rarefaction curves confirmed OTU saturation for most of the sequences (S2 Fig). In all four mammals, the most abundant bacterial order was Bacteroidales (52.07% +/- 3.02% (porcupine), 58.20% +/- 5.61% (beaver), 34.64% +/- 13.68% (coyote), and 37.96% +/- 12.89% (Arctic wolf). The second most abundant order in the Arctic wolf and porcupine was Clostridiales (30.38% +/- 16.78%, and 30.85% +/- 4.19%, respectively), whereas in the coyote it was Fusobacteriales (24.31% +/- 5.41%), and in the beaver it was unclassified (18.19% +/- 1.31%). A summary of the 16 highest orders in each mammalian sample can be found in Table 4, while the complete taxonomic assignment can be found in S2 Table.

**Table 4:** Taxonomic assignments for replicate sequencing of the Arctic wolf, coyote, beaver and porcupine. The 16 most abundant taxonomic assignments are shown for each mammal. All taxonomic assignments are average percent abundance. Standard deviation (StDev) is shown in brackets of each calculation.

Bacterial Order	Arctic Wolf Average (StDev)	Coyote Average (StDev)	Porcupine Average (StDev)	Beaver Average (StDev)
Bacteroidales	38.0 (+/-12.9)	34.6 (+/-13.7)	52.1 (+/-3.0)	58.2 (+/-5.6)
Clostridiales	30.4 (+/-16.8)	19.6 (+/-16.1)	30.9 (+/-4.2)	12.8 (+/-5.3)
Fusobacteriales	20.9 (+/-8.3)	24.3 (+/-5.4)	0	2.6 (+/-1.9)
Unassigned	7.34 (+/-1.8)	18.6 (+/-2.8)	10.4 (+/-0.9)	18.2 (+/-1.3)
Burkholderiales	0.97 (+/-0.5)	0.2 (+/-0.1)	0.1 (+/-0.1)	1.9 (+/-1)
Coriobacteriales	0.77 (+/-0.5)	1.3 (+/-1.5)	0.1	0.3 (+/-0.2)
Pseudomonadales	0	0	1.5 (+/-0.9)	0.2 (+/-0.2)
Erysipelotrichales	0.97 (+/-0.5)	0.9 (+/-0.6)	0.8 (+/-0.1)	1.5 (+/-1)
Aeromonadales	0.26 (+/-0.1)	0.3 (+/-0.2)	0	0.1 (+/-0.1)
RF39	0	0	0.9 (+/-0.5)	0.2 (+/-0.1)
Flavobacteriales	0	0	0.9 (+/-0.9)	0
YS2	0	0	0.4 (+/-0.1)	0
Enterobacteriales	0.1 (+/-0.1)	0	0.3 (+/-0.2)	0
Lactobacillales	0.2 (+/-0.1)	0	0.2 (+/-0.2)	0
Verrucomicrobiales	0	0	0.9 (+/-0.9)	3.5 (+/-0.7)
RF32	0	0	0.2 (+/-0.1)	0

Next the genetic repertoire of the microbiome was analyzed using metagenomic sequencing. The porcupine, beaver, coyote, and Arctic wolf samples were sequenced in triplicate to allow statistical analysis. Following sequencing, the mean number of reads were 4,378,272 for the porcupine; 4,599,146 for the beaver; 4,208,902 for the coyote; and 4,137,256 for the Arctic wolf. After stitching, more than 50% of sequences remained in each sample, and after screening, greater than 43.5% of the original sequences remained (Table 5).

**Table 5:** Summary of profiled species and sequences from shotgun metagenomic sequencing. Presented is the number of reads in total, after stitching with PEAR, and after screening to remove human and PhiX reads.

Host Animal	Replicate	Number of Reads	Reads After Stitching	Reads After Screening/Trimming
**Arctic Wolf (***Canis lupus* subs. *arctos*)	1	4,588,281	3,436,267	3,134,724
	2	2,760,442	1,721,568	1,655,754
	3	5,063,044	4,018,295	3,247,880
**Coyote** **(***Canis latrans*)	1	4,724,246	3,312,867	3,177,125
	2	3,152,637	1,617,376	1,563,285
	3	4,749,822	3,553,397	3,245,236
**Beaver** **(***Procyon lotor*)	1	4,597,577	3,233,205	3,053,519
	2	4,491,180	3,018,441	2,880,856
	3	4,708,681	3,641,930	3,266,187
**Porcupine (***Erethizon dorsatum*)	1	3,534,138	2,243,918	2,170,701
	2	4,796,618	3,489,595	3,311,107
	3	4,805,060	3,711,043	3,256,940

**Fig 3:**
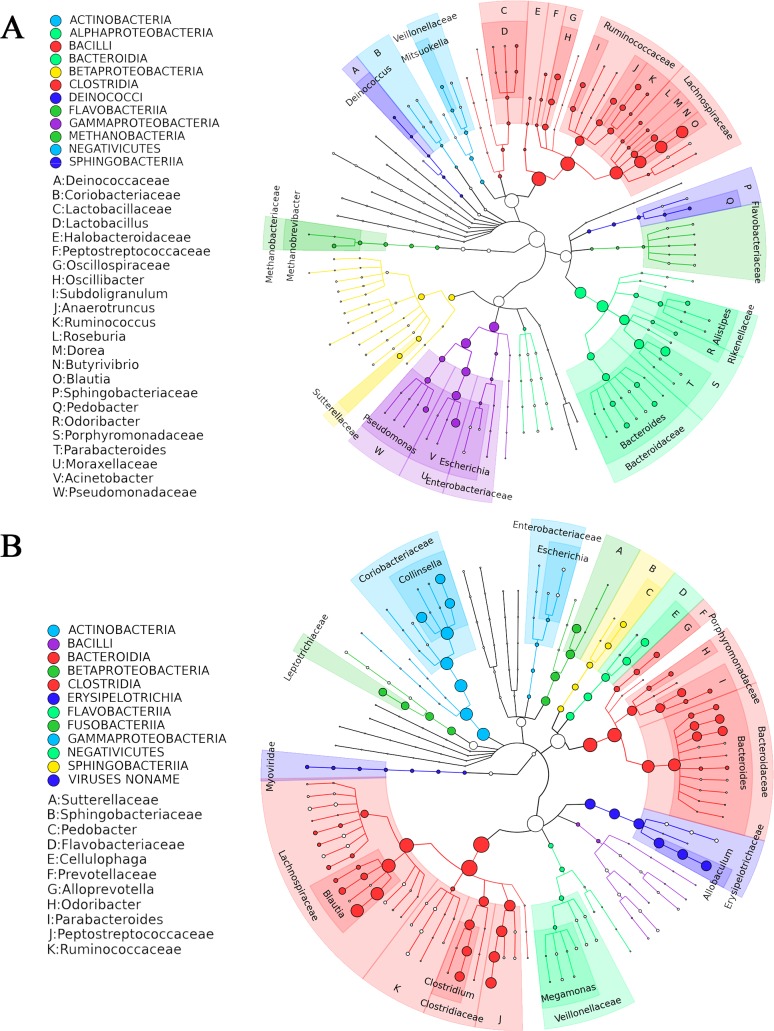
Metagenomic taxonomic summary of select Rodentia (porcupine and beaver) and Carnivora mammals (coyote and Arctic wolf). Each dot represents a node in the phylogenetic tree, whose size indicates the prevalence of representatives of that node in the sample. Colours are used to group bacteria into orders, indicated in each panel. Species identified with high abundance are labelled (A through W, and A through K in Panels A and B, respectively), while some with low abundance are not. Both the Rodentia mammals (A) and the Carnivora mammals (B) are represented.

Further taxonomic characterization of the porcupine, beaver, coyote, and Arctic wolf microbiomes was conducted by analyzing the metagenomic shotgun sequencing data. The microbial taxonomy of the beaver and porcupine microbiomes were similar to each other but diverged from microbiome taxonomies of the Arctic wolf and coyote (Fig 3). Perhaps not surprisingly, the most prevalent bacteria in both groups were Clostridiales (49.46% and 38.78% in the cellulose-consuming and carnivorous groups, respectively) and Bacteroidales (22.77% and 21.08% respectively). In the cellulose-consumer group, the next most prevalent bacterial orders were: Pseudomonales (13.22%), Burkholderiales (3.99%), Methanobacteriales (1.49%), Selenomonadales (1.42%), and Sphingobacteriales (1.26%). In the carnivorous group, the next most prevalent bacterial orders were: Coriobacteriales (14.49%), Erysipelotrichales (7.82%), Burkholderiales (4.94%), Fusobacteriales (4.99%), and Flavobacteriales (3.72%). Apart from the most prominent bacteria (Clostridiales and Bacteroidales) and the least prominent (Burkholderiales) that appear in both the carnivore and herbivore groups, bacterial composition is distinct. The complete taxonomic assignment for the metagenomic shotgun sequencing can be found in S3 Table.

### Cellulose metabolism genes are more abundant in hind-gut fermenters than carnivores

Next, the gene family profile was annotated using HUMAnN1 to determine the abundances of KEGG pathways in the four select mammalian samples. The highest mean pathway abundances in the Arctic wolf, coyote, beaver and porcupine samples were: D-glutamine and D-glutamate metabolism (KO00471; 3.27%, 3.16%, 2.90%, 2.73%), ribosome (KO03010; 2.89%, 2.87%, 2.81%, 3.09%), aminoacyl-tRNA biosynthesis (KO00970; 2.50%, 2.39%, 2.45%, 2.71%), valine, leucine and isoleucine biosynthesis (KO00290; 2.43%, 2.32%, 2.81%, 3.03%) and one-carbon pool by folate (KO00670; 2.36%, 2.18%, 2.32%, 2.24%) (Table 6). These pathways were expected to be among the highest as they are core metabolic pathways [43]. Complete functional assignments for metagenomic shotgun sequencing can be found in S4 Table.

**Table 6:** The 13 most abundant gene pathways identified using HUMAnN1 (metagenomic sequencing) in the Arctic wolf, coyote, porcupine, and beaver. Units are relative abundance of gene pathway sequences expressed as a percentage average. Standard deviation (StDev) is shown in brackets.

Gene Pathway (KEGG)	Arctic Wolf Mean(StDev)	Coyote Mean(StDev)	Porcupine Mean(StDev)	Beaver Mean(StDev)
D-Glutamine and D-glutamate metabolism	3.27(+/-0.16)	3.16 (+/-0.12)	2.73 (+/-0.14)	2.9 (+/-0.05)
Ribosome	2.89(+/-0.21)	2.87 (+/-0.26)	3.09 (+/-0.05)	2.81 (+/-0.09)
Valine, leucine and isoleucine biosynthesis	2.43(+/-0.16)	2.32 (+/-0.13)	3.03 (+/-0.05)	2.81 (+/-0.13)
Aminoacyl-tRNA biosynthesis	2.5(+/-0.22)	2.39 (+/-0.2)	2.71 (+/-0.06)	2.45 (+/-0.13)
One carbon pool by folate	2.36(+/-0.08)	2.18 (+/-0.1)	2.24 (+/-0.03)	2.32 (+/-0.09)
Thiamine metabolism	2.23(+/-0.05)	2.11 (+/-0.02)	1.74 (+/-0.03)	2.05 (+/-0.04)
Peptidoglycan biosynthesis	2.18(+/-0.07)	2.16 (+/-0.09)	2.03 (+/-0.04)	1.98 (+/-0.03)
Alanine, aspartate and glutamate metabolism	2.13(+/-0.06)	2.06 (+/-0.1)	2.19 (+/-0.06)	2.22 (+/-0.13)
Lysine biosynthesis	1.87(+/-0.09)	1.78 (+/-0.03)	1.93 (+/-0.02)	2.01 (+/-0.01)
Streptomycin biosynthesis	1.67(+/-0.1)	1.77 (+/-0.03)	2.1 (+/-0.23)	2 (+/-0.9)
Biotin metabolism	1.89(+/-0.61)	2.09 (+/-0.5)	1.33 (+/-0.11)	1.67 (+/-0.15)
D-Alanine metabolism	1.82(+/-0.2)	1.87 (+/-0.07)	2.73 (+/-0.06)	1.83 (+/-0.08)
Pantothenate and CoA biosynthesis	1.75(+/-0.02)	1.77 (+/-0.1)	1.97 (+/-0.07)	2.01 (+/-0.03)

**Fig 4:**
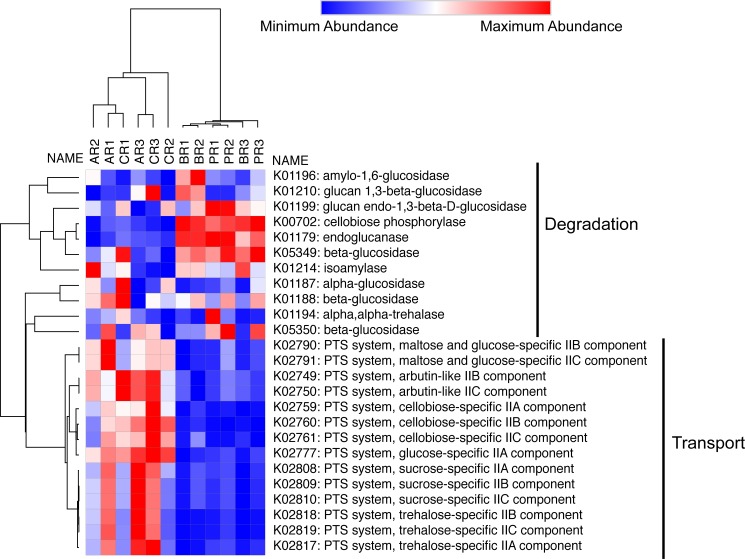
Summary functional heat-map of select enzymes in the starch and sucrose metabolism KEGG pathway. Functional assignment of the metagenomic sequencing was done using HUMAnN and the KEGG database. Generation of the above heatmap was done using Morpheus (see methods) using only genes in the starch and sucrose metabolism pathway, which has been simplified for clarity. Transport and degradation genes in the starch and sucrose metabolism pathway are labelled with black lines. Rows and columns were clustered using the hierarchal clustering tool in Morpheus, using the one minus Pearson correlation matrix and the average linking method. AR, Arctic wolf replicate; CR, coyote replicate; BR, beaver replicate; PR, porcupine replicate.

A heat-map was then generated to visualize the starch and sucrose pathway families identified from the KEGG database. Here cellulolytic and sugar transport genes were displayed from the metagenomic data from the four animals (Fig 4). The map demonstrated that the porcupine and the beaver had high abundances of cellulose-metabolizing genes and low abundances of transport genes. The opposite result was true for the coyote and Arctic wolf.

**Fig 5:**
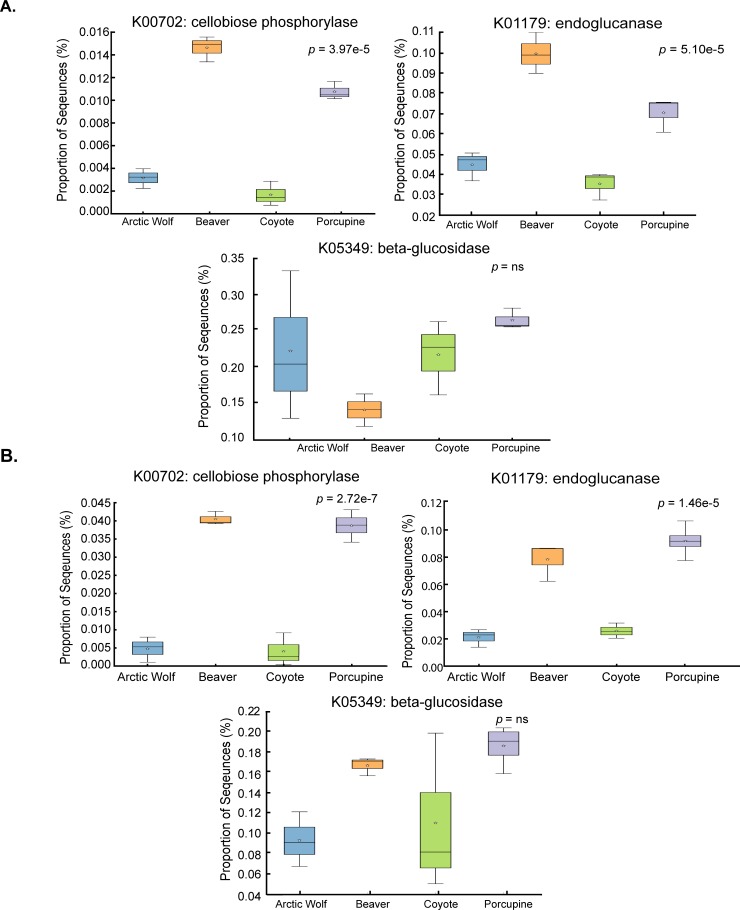
The comparison between the predicted (PICRUSt) and actual (HUMAnN1) mean sequence proportion of genes encoding particular enzymes in selected mammalian microbiomes. Box plots of predicted (**A; PICRUSt**) or identified (**B; HUMAnN1**) mean sequence proportion of genes encoding the indicated enzymes are shown for feces of four indicated mammals. Predicted data were obtained via PICRUSt analysis from 16S rRNA sequencing, in triplicate. Mean sequence proportions were obtained via HUMAnN1 analysis from shotgun metagenomic sequencing, in triplicate. Statistical significance was determined using ANOVA with Benjamini-Hochberg FDR correction for multiple tests.

Three cellulose-metabolizing genes were identified from the heat map and selected to compare relative gene abundances in the porcupine, beaver, coyote, and Arctic Wolf. The three representative enzymes were: endoglucanase (K01179), cellobiose phosphorylase (K00702) and beta-glucosidase (K05349). These enzymes were used based on their critical role in cellulose metabolism. PICRUSt analysis (using 16S sequencing) predicted a significant enrichment of the mean relative frequencies of endoglucanase and cellobiose phosphorylase in the beaver (0.099%, and 0.015%) and porcupine (0.070%, and 0.011%), in comparison to the Arctic wolf (0.044%, and 0.003%) and coyote (0.035%, and 0.002%) (Fig 5A, p = 5.10e-5, and p = 3.97e-5, respectively). The predicted mean relative frequency of beta-glucosidase was not statistically significant (Arctic wolf (0.21%), coyote (0.23%), beaver (0.14%) and porcupine (0.27%, Fig 5A). Metagenomic analysis was concordant with most of the PICRUSt predictions. For endoglucanase and cellobiose phosphorylase, there was a significant enrichment of mean relative frequencies in the beaver (0.0781%, and 0.0406%), and porcupine (0.0388%, and 0.0915%), in comparison to the Arctic wolf (0.0212%, and 0.0046%) and coyote (0.0039%, and 0.0259%) (Fig 5B, p = 1.46e-5, and p = 2.72e-7, respectively). The predicted mean relative frequency of beta-glucosidase was not statistically significant (Arctic wolf (0.00093%), beaver (0.0017%), coyote (0.0011%), and porcupine (0.0019%) (Fig 5B).

**Fig 6:**
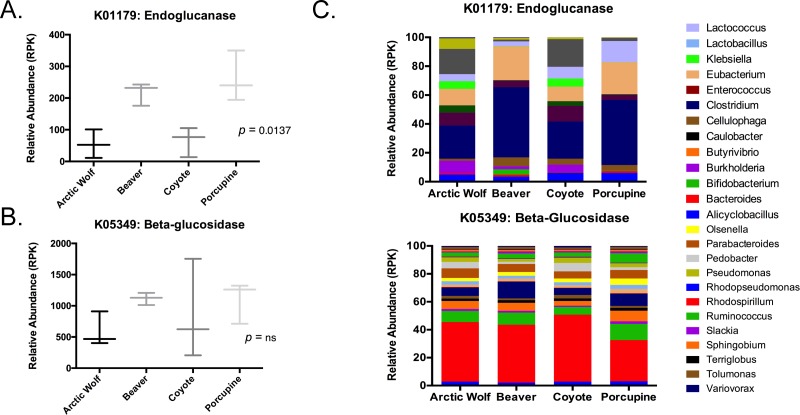
HUMAnN2 analysis gene family abundance and these gene families contributing taxa. Whisker plots for the relative abundance (measured in reads mapped per kilobase) were generated from HUMAnN2 functional analysis for endoglucanase (K01179) and beta-glucosidase (K05349). A two-way ANOVA using Holm-Sidak multiple test correction we used to test significance (A, *p* = 0.0137; B, not significant). The taxonomic breakdown for each gene family was also generated using HUMAnN2 output and presented at the genus level (C).

HUMAnN2 analysis was subsequently used to identify the relative abundance of endoglucanase and beta-glucosidase in the four select mammals (cellobiose phosphorylase was not annotated when using HUMAnN2). The average relative abundance (per kb) of endoglucanase (K01179) was 56.23, 63.83, 216.97, and 303.6 for the Arctic wolf, coyote, beaver, and porcupine, respectively (Fig 6, *p* = 0.0137). The average relative abundance (per kb) of beta-glucosidase (K05349) was 593.16, 861.17, 1116.26 and 1098.92 for the Arctic wolf, coyote, beaver, and porcupine, respectively (Fig 6, *p* = ns). HUMAnN2 enables quantification of taxonomic orders that contribute to enzyme abundance. Here, HUMAnN2 analysis was used to provide a taxonomic breakdown of organisms likely contributing to beta-glucosidase and endoglucanase abundance in the porcupine, beaver, coyote, and Arctic wolf microbiomes (S5 Table). The most abundant organisms contributing the beta-glucosidase gene family to the Arctic wolf, coyote, beaver, and porcupine microbiomes were *Bacteroides* (42.66%, 47.89%, 41.48%, 30.55%), *Bifidobacterium* (8.05%, 5.61%, 8.72%, 11.54%), *Clostridium* (5.84%, 4.85%, 11.96%, 8.55%), *Parabacteroides* (6.86%, 5.08%, 5.80%, 6.29%), and *Butyrivibrio* (5.72%, 3.27%, 5.74%, 7.35%) (Table 7). The most abundant organisms contributing the endoglucanase gene family to the Arctic wolf, coyote, beaver, and porcupine microbiomes were *Clostridium* (22.95%, 25.83%, 48.66%, 42.82%), *Eubacterium* (11.51%, 10.05%, 23.52%, 24.93*%)*, *Methanobrevibacter* (17.49%, 19.13%, 1.23%, 2.18%), *Lactococcus* (4.96%, 8,19%, 3.25%, 13.73%) and *Deinococcus* (9.00%, 10.77%, 4.55% and 3.28%) (Table 8). The entirety of the HUMAnN2 dataset can be found in S5 Table.

**Table 7:** Average percent abundances of bacterial genus’ contributing beta-glucosidase (K05349) in the Arctic wolf, coyote, beaver and porcupine microbiomes as analyzed by HUMAnN2. Average percent abundances were obtained by calculating the percentage of each organism in each run (relative abundance divided by total abundance) and then taking the average of these means. Each sample was sequenced in biological triplicate times, except the porcupine which was sequenced in biological quadruplicate.

K05349: Beta-glucosidase	Arctic Wolf	Coyote	Beaver	Porcupine
*Bacteroides*	42.66%	47.89%	41.48%	30.55%
*Bifidobacterium*	8.05%	5.60%	8.72%	11.54%
*Clostridium*	5.84%	4.85%	11.96%	8.55%
*Parabacteroides*	6.86%	5.08%	5.80%	6.29%
*Butyrivibrio*	5.72%	3.27%	5.74%	7.35%
*Ruminococcus*	3.02%	3.04%	3.44%	6.54%
*Pedobacter*	4.59%	6.03%	1.59%	2.01%
*Pseudomonas*	3.39%	3.83%	2.07%	2.98%
*Olsenella*	2.31%	2.70%	2.62%	4.34%
*Alicyclobacillus*	2.79%	2.85%	2.12%	3.06%
*Lactobacillus*	2.40%	2.13%	1.98%	2.75%
*Eubacterium*	1.70%	1.73%	1.97%	2.96%
*Caulobacter*	1.77%	1.87%	1.92%	1.98%
*Cellulophaga*	1.86%	2.43%	1.20%	1.36%
*Tolumonas*	1.56%	1.20%	1.77%	1.55%
*Burkholderia*	1.35%	0.94%	1.23%	1.76%
*Sphingobium*	1.25%	1.20%	1.21%	1.15%
*Slackia*	0.71%	0.93%	1.46%	1.25%
*Variovorax*	0.75%	1.07%	0.62%	0.72%
*Enterococcus*	0.54%	0.38%	0.26%	0.41%
*Terriglobus*	0.38%	0.30%	0.39%	0.42%
*Rhodopseudomonas*	0.18%	0.38%	0.25%	0.24%
*Rhodospirillum*	0.26%	0.27%	0.13%	0.16%
*Lactococcus*	0.06%	0.05%	0.06%	0.06%
*Klebsiella*	0.00%	0.00%	0.03%	0.00%

**Table 8:** Average percent abundances of bacterial genus’ contributing to endoglucanase (K01179) in the Arctic Wolf, Coyote, Beaver and Porcupine microbiomes as analyzed by HUMAnN2. Average percent abundances were obtained by calculating the percentage of each organism in each run (Relative abundance divided by total abundance) and then taking the average of these means. Each sample was sequenced in biological triplicate.

K01179: Endoglucanase	Arctic Wolf	Beaver	Coyote	Porcupine
*Clostridium*	22.95%	48.66%	25.83%	42.82%
*Eubacterium*	11.51%	23.52%	10.06%	24.93%
*Methanobrevibacter*	17.49%	1.23%	19.13%	2.18%
*Lactococcus*	4.96%	3.25%	8.19%	13.73%
*Deinococcus*	9.00%	4.55%	10.77%	3.28%
*Alicyclobacillus*	4.98%	3.54%	6.14%	6.87%
*Burkholderia*	8.67%	2.08%	5.13%	0.17%
*Cellulophaga*	1.47%	6.11%	4.14%	3.56%
*Klebsiella*	5.23%	0.17%	5.59%	0.15%
*Pseudomonas*	7.31%	1.08%	1.20%	0.17%
*Escherichia*	4.97%	0.22%	3.25%	0.43%

## Discussion

Here we report high abundance of cellulolytic enzymes in the porcupine gut microbiome. We initially surveyed 21 mammalian microbiomes using 16S rRNA sequencing, and clustered them into taxonomic groups (Fig 1). From this, we identified differences in bacterial composition amongst mammalian orders. We focused our attention on four mammals for in-depth 16S analysis in triplicate: the porcupine and beaver (Rodentia), and the coyote and Arctic wolf (*Canidae*). Clostridiales and Bacteroidales were abundant in both the Rodentia and *Canidae* groups. In addition, the Arctic wolf and coyote samples contained *Fusobacterium* and Burkholderiales, and the beaver and porcupine contained RF39 and Verrucomicrobiales. We observed that not all species grouped within their particular order (ex. the groundhog was separate from the rest of the Rodentia order), which may be in part due to the constraints of 16S rRNA sequencing. If we had selected a different 16S rRNA region, or had a larger database of known microorganisms, we may have seen an increased congruence of microbiomes amongst these animals [44]. However, without further sequencing of these microbiomes, we are unable to make any formal conclusions.

We re-sequenced the Arctic wolf, coyote, porcupine, and beaver in biological triplicate via metagenomic shotgun sequencing. Within the Rodentia mammals, Clostridiales and Bacteroidales still had the highest abundance, but Burkholderiales and Pseudomonadales were also prevalent. The Pseudomonadales are known to denitrify organic matter, and are part of the carbohydrate-metabolizing Gammaproteobacteria phylum [45]. A few examples of cellulose-metabolizing Gammaproteobacteria are *Acinetobacter*, *Pseudomonas*, and *Staphylococcus*, which have been isolated from termite gut microbiomes and have been shown to grow when cellulose is the sole carbon source [46]. Our metagenomic sequencing data did not precisely match our 16S rRNA sequencing data; we reasoned that our metagenomic sequencing data was likely more accurate due to previously reported biases in 16S primer sets [47]. In the *Canidae* mammals, the most abundant bacterial orders were Clostridiales and Bacteroidales, followed by Coriobacteriales, Erysipelotrichales and Burkholderiales. The roles of specific bacterial families in the *Canidae* gut microbiome may be inferred from human microbiome studies. For example, the *Coriobacteraceae* family (of the Coriobacteriales order) consists of bacteria that degrade bile salts and steroids [48] and the *Erysipelotrichaceae* family (of the Erysipelotichales order) have been linked to lipidemic profiles of human hosts [49]. Thus, the high prevalence of *Erysipelotrichaceae* in *Canidae* may be attributed to high fat diets.

Beavers and porcupines are monogastric hindgut fermenters that rely on symbiotic gut bacteria to break down plant material. Fermentation is restricted to the cecum in the porcupine, whereas the beaver uses both its cecum and the proximal colon [20]. Both animals feed on tree bark and similar plant material which likely explains certain similarities between their microbiomes, notably including high levels of known cellulose-degrading bacteria. Gruninger, *et*. *al*. recently sequenced the beaver microbiome from cecum and fecal samples, examining the microbial differences at the phylum level [10]. They found that the cecum and fecal microbiomes were both dominated by Bacteroidetes and Firmicutes. Our study did not sample from the beaver cecum, but we also found abundant Bacteroidales (class of Bacteroidetes) and Clostridiales (class of Firmicutes) bacteria in the fecal microbiome. Because porcupines and beavers have enlarged cecum’s housing complex microbial communities, we speculate that the beaver cecum microbiome would also be dominated by Bacteroidetes and Firmicutes. Unfortunately, V1-V3 primers were used to sequence the 16S rRNA in the Gruninger *et*. *al*. study, preventing a direct comparison between our datasets.

The functional diversity of the *Canidae* and *Rodentia* microbiomes can be attributed to distinct diets of carnivores and herbivores. Diet, in association with phylogeny, drives the evolution of mammalian gut microbiota [50] to the point that species-specific microbiomes share metabolic pathways with other animals that eat similar diets [51]. In this study, analysis of mammalian microbiomes might be considered biased as we are not sampling wild mammals, but instead park mammals that are fed a consistent diet based on species-specific nutritional requirements. The influence of captivity on microbial diversity remains poorly understood [11, 50, 52]. In studies in which captivity appeared to affect diversity, captive animals were fed a diet that differed from what they would experience in the wild [50, 53]. In our study, we observed low deviation between sample replicates (Fig 5) indicating that the microbiomes of both wild and captive animals are similar and stable (an observation similar to that made by Song et al. in cohabiting human microbiomes [54–55]).

Beyond taxonomic analysis, we compared the cellulose-degradation potential of the Arctic wolf, coyote, beaver, and porcupine microbiomes. To do so we measured starch and sucrose metabolism pathway abundance (which contains the cellulose metabolism pathway) [56]. We found that the porcupine and beaver microbiomes contained large abundances of cellulose metabolism genes (as expected) [10, 14, 57, 58], but low abundances of sugar transporter genes. The opposite result was observed in the Arctic wolf and coyote microbiomes. This result may be due to the physiological differences between the digestive tracts of herbivores, especially hind-gut fermenters, and carnivores [59]. The major feature differentiating these two groups is the cecum, which is located at the start of the large intestine [59]. In comparison to carnivores, hind-gut fermenters have a working, enlarged cecum which acts as the site of microbial digestion and fermentation [59]. This means that in hind-gut fermenters, the majority of food passes through the small intestine undigested. Hind-gut fermentation enables the breakdown of cellulose-rich plant material, but does not support uptake of nutrients, as reflected in our results (high abundances metabolizing genes, but low abundances of transporters) [59]. For this reason, hind-gut fermenters have evolved a number of behavioral and physical adaptations, such as eating shed feces, to maximize nutrient absorption [59]. Alternatively, carnivores rely on the small intestine, which is replete with carbohydrate transporters, for digestion and absorption of nutrients (as reflected in our results) [59].

Next, we selected three genes involved in cellulose crystal metabolism, di- and tri-saccharide subunit metabolism, and sugar utilization (endoglucanase, beta-glucosidase and cellobiose phosphorylase, respectively). We identified that the porcupine and beaver had significantly increased abundances of endoglucanase, and cellobiose phosphorylase (Fig 5). The abundance of endoglucanase in the porcupine and beaver were corroborated by PICRUSt, HUMAnN1, and HUMAnN2 analysis (Figs 5 and 6). HUMAnN2 analysis also provided a taxonomic breakdown of organisms, such as *Rhodospirillum* and *Cellulophaga*, likely contributing beta-glucosidase and endoglucanase in the porcupine, beaver, Arctic wolf, and coyote microbiomes (Fig 6C). Although *Bacteroides* are abundant in the beta-glucosidase functional assignment for each animal, *Clostridium* are responsible for roughly a two-fold increase of the enzyme in the beaver and porcupine in comparison to the coyote and Arctic wolf. This is to be expected, as *Clostridium* are often noted as important cellulose degraders [57]. For endoglucanase, *Eubacterium* and *Clostridium* play a nearly two-fold greater role in cellulosic breakdown in the herbivores, than in the Arctic wolf and coyote. This is perhaps not surprising, as the organisms that metabolize cellulose (like *Clostridium* spp.) are more abundant, thus there is a larger abundance of reads matching cellulose degradation.

Most of the enzymes needed to degrade complex plant polysaccharides are not present in mammalian genomes, resulting in a mutual dependence between the mammalian host and gut microbiota [58]. Zhu et al. investigated the bacterial diversity of the giant panda (*Ailuropoda melanoleuca)* which possesses a typical carnivore gastrointestinal tract, but which also consumes roughly 12.5 kg of bamboo each day [60]. Using 16S rRNA sequencing and gene function classification they identified cellulose-metabolizing bacteria in the panda microbiome. Separate groups have identified the cellulolytic potential of other microbiomes such as beavers [10], ruminants [14], and termites [61]. In our datasets, the porcupine and beaver had statistically higher abundances of endoglucanases and cellobiose phosphorylases than carnivores. Thus, mining animal gut microbiomes could provide a potentially rich source of novel microbial genes that could be leveraged for bioengineering applications.

Metagenomic libraries derived from hind-gut fermenter fecal samples could accelerate the discovery of novel cellulolytic enzymes using an approach known as synthetic metagenomics [62–63]. This technique chemically synthesizes selected genes of interest identified from functional metagenomic screens [63]. Synthetic metagenomics enables the identification and synthesis of thousands of novel enzyme sequences from complex environmental isolates. Combining traditional metagenomic library analysis with new synthetic metagenomics approaches will undoubtedly accelerate efforts to “mine the microbiome” of mammals of interest.

## Supporting information

S1 Fig Alpha rarefaction curve of the 21 mammal microbiomes sequenced by 16S rRNA sequencing. All animals sequenced in the initial microbiome survey are included above. Each line represents the function of the observed number of OTUs over the sequences per sample.
(TIF)

S2 Fig Alpha rarefaction curve of the replicate mammal microbiomes sequenced by 16S rRNA sequencing. The arctic wolf, beaver, coyote and porcupine, all which were sequenced in triplicate, are represented above. Each line represents the function of the observed number of OTUs over the sequences per sample.
(TIF)

S1 Table 16S taxonomy for all 21 mammals at Shubenacadie Wildlife Park. (XLSX)

S2 Table 16S taxonomy for the 4 mammals (Arctic Wolf, Coyote, Beaver, Porcupine) sequenced in triplicate at Shubenacadie Wildlife Park. (XLSX)

S3 Table MetaPhlAn taxonomic assignment data from metagenomic sequencing for the Arctic Wolf, Coyote, Beaver and Porcupine. (XLSX)

S4 Table HUMAnN functional assignment data from metagenomic sequencing for the Arctic Wolf, Coyote, Beaver and Porcupine. (XLSX)

S5 Table HUMAnN2 functional gene family assignment data from metagenomic sequencing for the Arctic Wolf, Coyote, Beaver and Porcupine. (ZIP)

## References

[1] Dunn KA, Moore-Connors J, MacIntyre B, Stadnyk A, Thomas NA, Noble A, et al The gut microbiome of pediatric Crohn’s disease patients differs from healthy controls in genes that can influence the balance between a healthy and dysregulated immune response. Inflamm Bowel Dis. 2016;22(11): 2607–2609. Available from: https://www.ncbi.nlm.nih.gov/pubmed/27760077. doi: 10.1097/MIB.0000000000000949 27760077 10.1097/MIB.0000000000000949

[2] The Human Microbiome Project Consortium. Structure, function and diversity of the healthy human microbiome. Nature. 2012;486(7402): 207–210. Available from: https://www.ncbi.nlm.nih.gov/pubmed/22699609. doi: 10.1038/nature11234 22699609 10.1038/nature11234 PMC3564958

[3] Turnbaugh PJ, Ley RE, Hamady M, Fraser-Liggett C, Knight R, Gordon JI. The human microbiome project: exploring the microbial part of ourselves in a changing world. Nature. 2007;449(7164): 804–810. Available from: https://www.ncbi.nlm.nih.gov/pmc/articles/PMC3709439/. doi: 10.1038/nature06244 17943116 10.1038/nature06244 PMC3709439

[4] The NIH Working Group, Peterson J, Garges S, Giovanni M, McInnes P, Wang L, et al The NIH human microbiome project. Genome Res. 2009;19(12): 2317–2323. Available from: https://www.ncbi.nlm.nih.gov/pubmed/19819907. doi: 10.1101/gr.096651.109 19819907 10.1101/gr.096651.109 PMC2792171

[5] Round JL, Mazmanian SK. The gut microbiome shapes intestinal immune responses during health and disease. Nat Rev Immunol. 2009;9(5): 313–320. Available from: https://www.ncbi.nlm.nih.gov/pmc/articles/PMC4095778/. doi: 10.1038/nri2515 19343057 10.1038/nri2515 PMC4095778

[6] Greenblum S, Turnbaugh PJ, Borenstein E. Metagenomic systems biology of the human gut microbiome reveals topological shifts associated with obesity and inflammatory bowel disease. Proc Natl Acad Sci USA. 2012;109(2): 594–599. Available from: https://www.ncbi.nlm.nih.gov/pubmed/22184244. doi: 10.1073/pnas.1116053109 22184244 10.1073/pnas.1116053109 PMC3258644

[7] David LA, Maurice CF, Carmody RN, Gootenberg DB, Button JE, Wolfe BE, et al Diet rapidly and reproducibly alters the human gut microbiome. Nature. 2014;505(7484): 559–563. Available from: https://www.ncbi.nlm.nih.gov/pubmed/24336217. doi: 10.1038/nature12820 24336217 10.1038/nature12820 PMC3957428

[8] Cho I, Blaser MJ. The human microbiome: at the interface of health and disease. Nat Rev Genet. 2012;13(4): 260–270. Available from: https://www.ncbi.nlm.nih.gov/pmc/articles/PMC3418802/. doi: 10.1038/nrg3182 22411464 10.1038/nrg3182 PMC3418802

[9] Qin N, Yang F, Li A, Prifti E, Chen Y, Shao L, et al Alternations of the human gut microbiome in liver cirrhosis. Nature. 2014; 513(7516): 59–60. Available from: https://www.ncbi.nlm.nih.gov/pubmed/25079328. doi: 10.1038/nature13568 25079328 10.1038/nature13568

[10] Gruninger RJ, McAllister TA, Forester RJ. Bacterial and archaeal diversity in the gastrointestinal tract of the North American beaver (*Castor canadensis*). PLoS One. 2016;11(5): e0156457 Available from: https://www.ncbi.nlm.nih.gov/pubmed/27227334. doi: 10.1371/journal.pone.0156457 27227334 10.1371/journal.pone.0156457 PMC4881982

[11] Ley RE, Hamady M, Lozupone C, Turnbaugh PJ, Ramey RR, Bircher JS, et al Evolution of mammals and their gut microbes. Science. 2008;320(5883): 1647–1651. Available from: https://www.ncbi.nlm.nih.gov/pubmed/18497261. doi: 10.1126/science.1155725 18497261 10.1126/science.1155725 PMC2649005

[12] Gulino L-M, Ouwerkerk D, Kang AYH, Maguire AJ, Kienzle M, Klieve AV. Shedding light on the microbial community of the macropod foregut using 454-amplicon pyrosequencing. PLoS One. 2013;8(4): e61463 Available from: http://journals.plos.org/plosone/article?id=10.1371/journal.pone.0061463. doi: 10.1371/journal.pone.0061463 23626688 10.1371/journal.pone.0061463 PMC3634081

[13] Gruninger RJ, Sensen CW, McAllister TA, Forster RJ. Diversity of rumen bacteria in Canadian cervids. PLoS One. 2014;9(2): e89682 Available from: https://www.ncbi.nlm.nih.gov/pubmed/24586961. doi: 10.1371/journal.pone.0089682 24586961 10.1371/journal.pone.0089682 PMC3937448

[14] Henderson G, Cox F, Ganesh S, Jonker A, Young W, Global Rumen Census Collaborators, et al Rumen microbial community composition varies with diet and host, but a core microbiome is found across a wide geographical range. Sci Rep. 2015;5: 14567 Available from: https://www.ncbi.nlm.nih.gov/pubmed/26449758. doi: 10.1038/srep14567 26449758 10.1038/srep14567 PMC4598811

[15] David LA, Maurice CF, Carmody RN, Gootenberg DB, Button JE, Wolfe BE, et al Diet rapidly and reproducibly alters the human gut microbiome. Nature. 2014;505(7484): 559–563. Available from: https://www.ncbi.nlm.nih.gov/pubmed/24336217. doi: 10.1038/nature12820 24336217 10.1038/nature12820 PMC3957428

[16] Zhu L, Wi Q, Dai J, Zhang S, Wei F. Evidence of cellulose metabolism by the giant panda gut microbiome. Proc. Natl. Acad. Sci. USA. 2010; 108(43): 17714 Available from: https://www.ncbi.nlm.nih.gov/pubmed/22006317. 10.1073/pnas.1017956108 PMC3203778 22006317

[17] Ravindran R, Jaiswal AM. Microbial enzyme production using lignocellulosic food industry wastes as feedstock: a review. Bioeng. 2016;3(4). doi: 10.3390/bioengineering3040030 Available from: http://www.mdpi.com/2306-5354/3/4/30/htm 28952592 10.3390/bioengineering3040030 PMC5597273

[18] Rubin EM. Genomics of cellulosic biofuel. Nature. 2008;454(7206): 841–845. Available from: https://www.ncbi.nlm.nih.gov/pubmed/18704079. doi: 10.1038/nature07190 18704079 10.1038/nature07190

[19] Graham D. Porcupine. South Dakota Department of Game, Fish and Parks, Division of Wildlife. Available from: http://www3.northern.edu/natsource/MAMMALS/Porcup1.htm.

[20] Vispo C, Hume ID. The digestive tract and digestive function in the North American porcupine and beaver. Can J Zool. 1995;73(5): 967–974. Available from: http://www.nrcresearchpress.com/doi/abs/10.1139/z95-113#.WaQs8hgZPG4.

[21] Choo JM, Leong LEX, Rogers GB. Sample storage conditions significantly influence faecal microbiome profiles. Nat Sci Rep. 2015;5(16350). doi: 10.1038/srep16350 Available from: https://www.nature.com/articles/srep16350. 26572876 10.1038/srep16350 PMC4648095

[22] Comeau AM, Li WKW, Tremblay J-É, Carmack ED, Lovejoy C. Arctic ocean microbial community structure before and after the 2007 record sea ice minimum. PLoS One. 2011;6(11): e27492 Available from: https://www.ncbi.nlm.nih.gov/pubmed/22096583. doi: 10.1371/journal.pone.0027492 22096583 10.1371/journal.pone.0027492 PMC3212577

[23] Comeau AM, Douglas GM, Langille MG. Microbiome Helper: A custom and streamlined workflow for microbiome research. mSystems. 2017 2(1). doi: 10.1128/mSystems.00127-16 Available from: https://www.ncbi.nlm.nih.gov/pmc/articles/PMC5209531/. 28066818 10.1128/mSystems.00127-16 PMC5209531

[24] Zhang J, Kobert K, Flouri T, Stamatakis A. PEAR: A fast and accurate illumina paired-end reAd mergeR. Bioinformatics. 2013;30(5): 614–20. Available from: https://www.ncbi.nlm.nih.gov/pubmed/24142950. doi: 10.1093/bioinformatics/btt593 24142950 10.1093/bioinformatics/btt593 PMC3933873

[25] Rognes T, Flouri T, Nichols B, Quince C, Mahé F. VSEARCH: a versatile open source tool for metagenomics. PeerJ. 2016;4:e2584 Available from: https://www.ncbi.nlm.nih.gov/pmc/articles/PMC5075697/pdf/peerj-04-2584.pdf doi: 10.7717/peerj.2584 27781170 10.7717/peerj.2584 PMC5075697

[26] McDonald D, Price MN, Goodrich J, Nawrocki EP, DeSantis TZ, Probst A, et al An improved Greengenes taxonomy with explicit ranks for ecological and evolutionary analyses of bacteria and archaea. ISME J. 2012;6(3): 610–18. Available from: https://www.ncbi.nlm.nih.gov/pmc/articles/PMC3280142/. doi: 10.1038/ismej.2011.139 22134646 10.1038/ismej.2011.139 PMC3280142

[27] Caporaso G, Kuczynski J, Stombaugh J, Bittinger K, Bushman FD, Costello EK, et al QIIME allows analysis of high-throughput community sequencing data. Nat Methods. 2010;7(5): 335–6. Available from: https://www.nature.com/nmeth/journal/v7/n5/full/nmeth.f.303.html. doi: 10.1038/nmeth.f.303 20383131 10.1038/nmeth.f.303 PMC3156573

[28] Love MI, Huber W, Anders S. Moderated estimation of fold change and dispersion for RNA-seq data with DESeq2. Genome Biol. 2014;15(12): 550 Available from: https://www.ncbi.nlm.nih.gov/pubmed/25516281. doi: 10.1186/s13059-014-0550-8 25516281 10.1186/s13059-014-0550-8 PMC4302049

[29] Lozupone C, Lladser ME, Knights D, Stombaugh J, Knight R. UniFrac: an effective distance metric for microbial community comparison. ISME J. 2011;5(2): 169–172. Available from: https://www.ncbi.nlm.nih.gov/pubmed/20827291. doi: 10.1038/ismej.2010.133 20827291 10.1038/ismej.2010.133 PMC3105689

[30] Kanehisa M, Furumichi M, Tanabe M, Sato Y, Morishima K. KEGG: new perspectives on genomes, pathways, diseases and drugs. Nucleic Acids Res. 2017;4(45). doi: 10.1093/nar/gkw1092 Available from: https://www.ncbi.nlm.nih.gov/pubmed/27899662. 10.1093/nar/gkw1092 PMC5210567 27899662

[31] Kanehisa M, Sato Y, Kawashima M, Furumichi M, Tanabe M. KEGG as a reference resource for gene and protein annotation. Nucleic Acids Res. 2016;4(44): doi: 10.1093/nar/gkv1070 Available from: https://www.ncbi.nlm.nih.gov/pubmed/26476454. 10.1093/nar/gkv1070 PMC4702792 26476454

[32] Kanehisa M, Goto S. KEGG: Kyoto encyclopedia of genes and genomes. Nucleic Acids Res. 2000;1(28): 27–30. Available from: https://www.ncbi.nlm.nih.gov/pmc/articles/PMC102409/. 10.1093/nar/28.1.27 PMC102409 10592173

[33] Langille MGI, Zaneveld J, Caporaso JG, McDonald D, Knights D, Reyes JA, et al Predictive functional profiling of microbial communities using 16S rRNA marker gene sequences. Nat Biotechnol. 2013;31(9): 814–821. Available from: https://www.ncbi.nlm.nih.gov/pubmed/23975157. doi: 10.1038/nbt.2676 23975157 10.1038/nbt.2676 PMC3819121

[34] Parks DH, Tyson GW, Hugenholtz P, Beiko RG. STAMP: statistical analysis of taxonomic and functional profiles. Bioinformatics. 2014;30(21): 3123–24. Available from: https://www.ncbi.nlm.nih.gov/pubmed/25061070. doi: 10.1093/bioinformatics/btu494 25061070 10.1093/bioinformatics/btu494 PMC4609014

[35] Langmead B, Salzberg S. Fast gapped-read alignment with Bowtie 2. Nat Methods 2012;9(4): 357–359. Available from: https://www.ncbi.nlm.nih.gov/pubmed/22388286. doi: 10.1038/nmeth.1923 22388286 10.1038/nmeth.1923 PMC3322381

[36] Bolger AM, Lohse M, Usadel B. Trimmomatic: a flexible trimmer for Illumina sequence data. Bioinformatics. 2014;30(15): 2114–2120. Available from: https://www.ncbi.nlm.nih.gov/pubmed/24695404. doi: 10.1093/bioinformatics/btu170 24695404 10.1093/bioinformatics/btu170 PMC4103590

[37] Segata N, Waldron L, Ballarini A, Narasimhan V, Jousson O, Huttenhower C. Metagenomic microbial community profiling using unique clade-specific marker genes. Nat Methods. 2012;9(8): 811–814. Available from: https://www.ncbi.nlm.nih.gov/pubmed/22688413. doi: 10.1038/nmeth.2066 22688413 10.1038/nmeth.2066 PMC3443552

[38] Buchfink B, Xie C, Huson DH. Fast and sensitive protein alignment using DIAMOND. Nat Methods. 2015;12(1): 59–60. Available from: https://www.ncbi.nlm.nih.gov/pubmed/25402007. doi: 10.1038/nmeth.3176 25402007 10.1038/nmeth.3176

[39] Abubucker S, Segata N, Goll J, Schubert AM, Izard J, Cantarel BL, et al Metabolic reconstruction for metagenomic data and its application to the human microbiome. PLoS Comput Biol. 2012;8(6): e1002358 Available from: https://www.ncbi.nlm.nih.gov/pubmed/22719234. doi: 10.1371/journal.pcbi.1002358 22719234 10.1371/journal.pcbi.1002358 PMC3374609

[40] The Broad Institute (US). Morpheus [Internet]. [cited 2017 Aug 27]. Available from: https://software.broadinstitute.org/morpheus/documentation.html

[41] Wesley-Hunt GD, Flynn JJ. Phylogeny of the carnivore: Basal relationships among the carnivoramorphans, and assessment of the position of ‘miacoidea’ relative to carnivore. J Syst Palaeontol. 2010;3(1): 1–28. Available from: http://www.tandfonline.com/doi/abs/10.1017/s1477201904001518.

[42] Fulton TL, Strobeck C. Molecular phylogeny of the Arctoidea (Carnivora): Effect of missing data on supertree and supermatric analyses of multiple gene data sets. Mol Phylogenet Evol. 2006;4(1): 165–181. Available from: https://www.ncbi.nlm.nih.gov/pubmed/16814570. 10.1016/j.ympev.2006.05.025 16814570

[43] Karlin S, Mrázek J. Predicted highly expressed genes of diverse prokaryotic genomes. J Bacteriol. 2000;182(18): 5238–5250. Available from: https://www.ncbi.nlm.nih.gov/pmc/articles/PMC94675/. 10960111 10.1128/jb.182.18.5238-5250.2000 PMC94675

[44] Soergel DA, Dey N, Knight R, Brenner SE. Selection of primers for optimal taxonomic classification of environmental 16S rRNA gene sequences. ISME J. 2012;6(7): 1440–1444. Available from: https://www.ncbi.nlm.nih.gov/pubmed/22237546 doi: 10.1038/ismej.2011.208 22237546 10.1038/ismej.2011.208 PMC3379642

[45] Liu D, Li M, Xi B, Zhao Y, Wei Z, Song C, et al Metaproteomics reveals major microbial players and their biodegradation functions in a large-scale aerobic compositing plant. Microb. Biotechnol. 2015;8(6): 950–960. Available from: http://onlinelibrary.wiley.com/doi/10.1111/1751-7915.12290/full. doi: 10.1111/1751-7915.12290 25989417 10.1111/1751-7915.12290 PMC4621448

[46] Pourramenzan Z, Ghezelbash GR, Romani B, Ziaei S, Hedayatkhah A. Screening and identification of newly isolated cellulose-degrading bacteria from the gut of xylophagous termite *Microcerotermes diversus* (Silvestri). Mikrobiologiia. 2012; 81(6): 796–802. Available from: https://www.ncbi.nlm.nih.gov/pubmed/23610931. 23610931

[47] Tremblay J, Singh K, Fern A, Kirton ES, He S, Woyke T, et al Primer and platform effects on 16S rRNA tag sequencing. Front Microbiol. 2015;6: 771 doi: 10.3389/fmicb.2015.00771 Available from: https://www.ncbi.nlm.nih.gov/pubmed/26300854. 26300854 10.3389/fmicb.2015.00771 PMC4523815

[48] Clavel T, Lepage P, Charrier C. The Prokaryotes In: Rosenberg E, Delong E, Thompson F, Lory S, Stackebrandt E, editors. The Family Coriobacteriaceae. New York: Springer; 2014 p.201–238.

[49] Kaakoush NO. Insight into the role of *Erysipelotrichaeceae* in the human host. Front Cell Infect Microbiol. 2015; 5(84): doi: 10.3389/fcimb.2015.00084 Available from: https://www.ncbi.nlm.nih.gov/pmc/articles/PMC4653637/. 26636046 10.3389/fcimb.2015.00084 PMC4653637

[50] Delsuc F, Metcalf JL, Wegener Parfrey L, Song SJ, González A, Knight R. Convergence of gut microbiomes in myrmecophagous mammals. Mol Ecol. 2014;23(6): 1301–1316. Available from: https://www.ncbi.nlm.nih.gov/pubmed/24118574. doi: 10.1111/mec.12501 24118574 10.1111/mec.12501

[51] Sanders JG, Beichman AC, Roman J, Scott JJ, Emerson D, McCarthy JJ. Baleen whales host a unique gut microbiome with similarities to both carnivores and herbivores. Nat. Commun. 2015;6(82850). doi: 10.1038/ncomms9285 Available from: https://www.nature.com/articles/ncomms9285. 26393325 10.1038/ncomms9285 PMC4595633

[52] Muegge BD, Kuczynski J, Knights D, Clemente JC, González A, Fontana L, et al Diet drives convergence in gut microbiome functions across mammalian phylogeny and within humans. Science. 2011;332(6032): 970–974. Available from: https://www.ncbi.nlm.nih.gov/pubmed/21596990. doi: 10.1126/science.1198719 21596990 10.1126/science.1198719 PMC3303602

[53] Clayton JB, Vangay P, Huang H, Ward T, Hillann BM, Al-Ghalith GA, et al Captivity humanizes the primate microbiome. Proc Natl Acad Sci USA. 2016;113(37): 10376–10381. Available from: https://www.ncbi.nlm.nih.gov/pubmed/27573830. doi: 10.1073/pnas.1521835113 27573830 10.1073/pnas.1521835113 PMC5027417

[54] Song SJ, Lauber C, Costello EK, Lozupone CA, Humphrey G, Berg-Lyons D. Cohabiting family members share microbiota with one another and with their dogs. eLife. 2013;2: e00458 Available from: https://www.ncbi.nlm.nih.gov/pmc/articles/PMC3628085/. doi: 10.7554/eLife.00458 23599893 10.7554/eLife.00458 PMC3628085

[55] Coyte KZ, Schluter J, Foster KR. The ecology of the microbiome: Networks, competition, and stability. Science. 2015;350(6261): 663–666. Available from: https://www.ncbi.nlm.nih.gov/pubmed/26542567. doi: 10.1126/science.aad2602 26542567 10.1126/science.aad2602

[56] Kanehisa Laboratories. Starch and Sucrose Metabolism. KEGG Pathway Map. 2017. Available from: http://www.genome.jp/kegg-bin/show_pathway?map=map00500&show_description=show

[57] Ren Z, War TE, Logan BE, Regen JM. Characterization of the cellulolytic and hydrogren-producing activites of six mesophilic *Clostridum* species. J Appl Microbiol. 2007;103(6): 2258–2266. Available from: https://www.ncbi.nlm.nih.gov/pubmed/18045409 doi: 10.1111/j.1365-2672.2007.03477.x 18045409 10.1111/j.1365-2672.2007.03477.x

[58] Flint HJ, Scott KP, Duncan SH, Louis P, Forano E. Microbial degradation of complex carbohydrates in the gut. Gut Microbes. 2012;3(4): 289 Available from: https://www.ncbi.nlm.nih.gov/pmc/articles/PMC3463488/. doi: 10.4161/gmic.19897 22572875 10.4161/gmic.19897 PMC3463488

[59] Karasov WH, Douglas AE. Comparative Digestive Physiology. Compr Physiol. 2013;3(2): 741–783. Available from: https://www.ncbi.nlm.nih.gov/pmc/articles/PMC4458075/ doi: 10.1002/cphy.c110054 23720328 10.1002/cphy.c110054 PMC4458075

[60] Zhu L, Wi Q, Dai J, Zhang S, Wei F. Evidence of cellulose metabolism by the giant panda gut microbiome. Proc. Natl. Acad. Sci. USA. 2011; 108(43): 17714 Available from: https://www.ncbi.nlm.nih.gov/pmc/articles/PMC3203778/. doi: 10.1073/pnas.1017956108 22006317 10.1073/pnas.1017956108 PMC3203778

[61] Brune A. Symbiotic digestion of lignocellulose in termite guts. Nat Rev Microbiol. 2014; 12(3): 168–180. Available from: http://www.nature.com/nrmicro/journal/v12/n3/abs/nrmicro3182.html. doi: 10.1038/nrmicro3182 24487819 10.1038/nrmicro3182

[62] Cheng J, Pinnell L, Engel K, Neufeld JD, Charles TC. Versatile broad-host-range cosmids for construction of high quality metagenomic libraries. J Microbiol Methods. 2014; 99: 27–34. Available from: https://www.ncbi.nlm.nih.gov/pubmed/24495694. doi: 10.1016/j.mimet.2014.01.015 24495694 10.1016/j.mimet.2014.01.015

[63] Culligan EP, Sleator RY, Marchesi JR, Hill C. Metagenomics and novel gene discovery. *Virulence*. 2014; 5(3): 399–412. doi: 10.4161/viru.27208 24317337 10.4161/viru.27208 PMC3979868

