# Supplementary figures and images for: Taxonomic differences of gut microbiomes drive cellulolytic enzymatic potential within hind-gut fermenting mammals
Source: PLoS One. 2017 Dec 27;12(12):e0189404. doi: 10.1371/journal.pone.0189404 (PMC5744928; doi:10.1371/journal.pone.0189404)

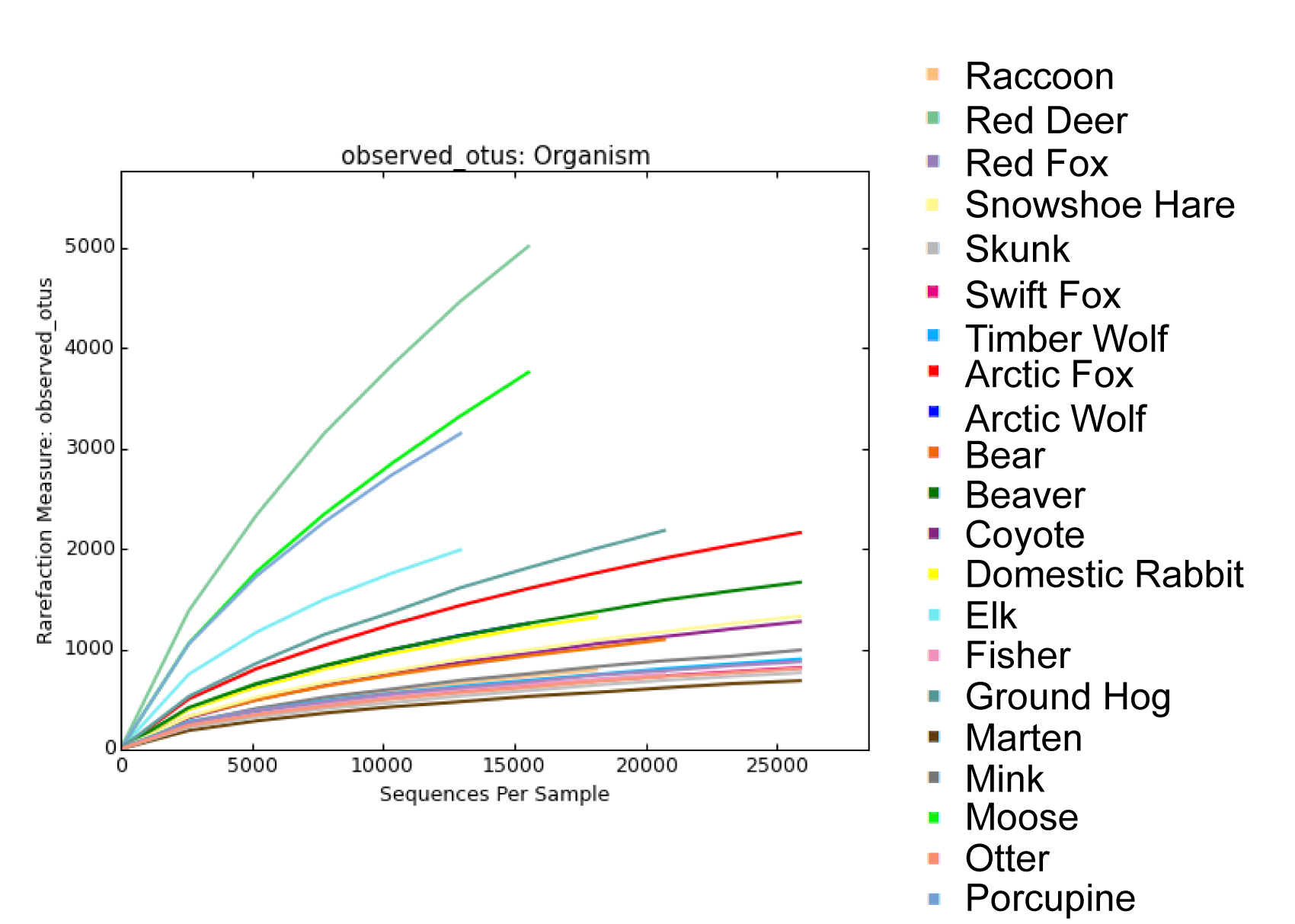

Supplement: S1 Fig — All animals sequenced in the initial microbiome survey are included above. Each line represents the function of the observed number of OTUs over the sequences per sample. (TIF) [file pone.0189404.s001.tif]

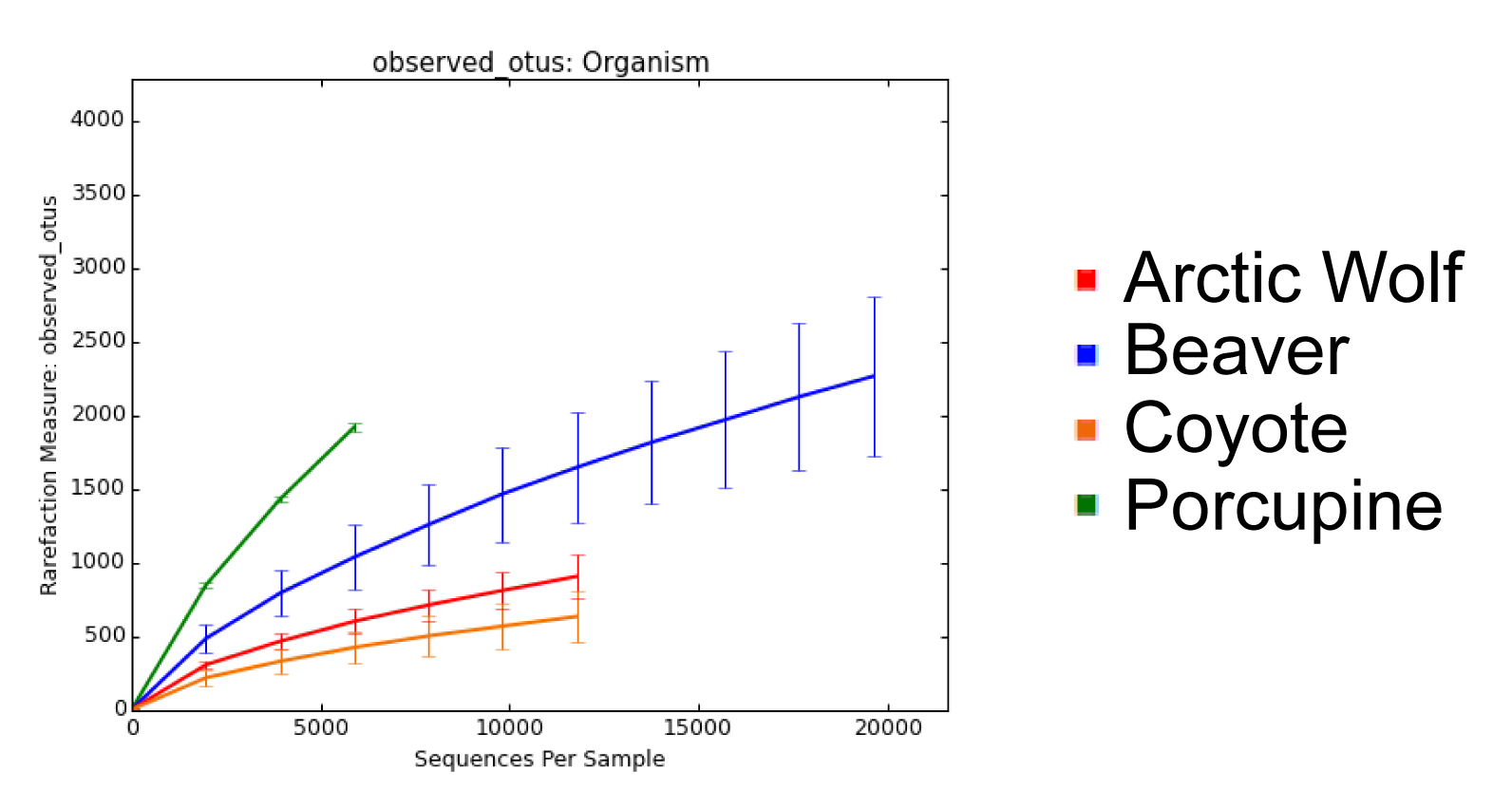

Supplement: S2 Fig — The arctic wolf, beaver, coyote and porcupine, all which were sequenced in triplicate, are represented above. Each line represents the function of the observed number of OTUs over the sequences per sample. (TIF) [file pone.0189404.s002.tif]
